# Central thromboxane A2, prostaglandin F2α, prostaglandin E, and prostaglandin D contribute to the cardiovascular effects elicited by nesfatin-1

**DOI:** 10.55730/1300-0144.5827

**Published:** 2024-03-27

**Authors:** Gökçen GÜVENÇ BAYRAM, Murat YALÇIN

**Affiliations:** 1 Department of Physiology, Faculty of Veterinary Medicine, Bursa Uludag University, Bursa, Turkiye; 2 Department of Physiology, Faculty of Veterinary Medicine, Dokuz Eylül University, Kiraz, İzmir, Turkiye

**Keywords:** Nesfatin-1, thromboxane A2, prostaglandin F2α, prostaglandin E, prostaglandin D, intracerebroventricular, mean arterial blood pressure, heart rate

## Abstract

**Background/aim:**

Our recent study revealed that the expression of lipoxygenase (LOX) and cyclooxygenase (COX) enzymes in the hypothalamus is activated by nesfatin-1, leading to the liberation of leukotrienes and prostaglandins (PG), respectively. Moreover, our prior report explained that intracerebroventricular (ICV) nesfatin-1 treatment triggers cardiovascular responses mediated by central LOX and COX enzymes. Building upon our prior reports, the present investigation sought to clarify the role of cardiovascularly active central COX products, such as thromboxane (TX) A2, PGF2α, PGE, and PGD, in orchestrating nesfatin-1-evoked reactions in mean arterial pressure (MAP) and heart rate (HR).

**Materials and methods:**

The Sprague Dawley rats, which had guide cannula in the lateral ventricle for intracerebroventricular (ICV) injections and catheter in arteria femoralis for monitoring MAP and HR, were underwent central pretreatment with furegrelate (the TXA2 synthase inhibitor), PGF2α-dimethylamine (PGF2α-DA, the PGF2α receptor antagonist), or AH6809 (the PGE and PGD receptor antagonist), 5 min prior to ICV nesfatin-1 administration. The cardiovascular parameters were observed and recorded for 60 min posttreatment

**Results:**

Nesfatin-1 induced cardiovascular responses in rats leading to pressor effect in MAP, and tachycardia following bradycardia in HR. Interestingly, ICV furegrelate, PGF2α-DA, or AH6809 pretreatment partially mitigated the cardiovascular effects revealed by nesfatin-1.

**Conclusion:**

The findings illuminate the role of nesfatin-1 in modulating MAP and HR through the central activation of specifically TXA2, PGF2α, PGE, and PGD from COX metabolites. Additionally, the study may also suggest the potential involvement of other central COX or LOX metabolites beyond these COX metabolites in mediating the cardiovascular effects produced by nesfatin-1.

## 1. Introduction

The brain robustly expresses nesfatin-1, initially identified as a hypothalamic neuropeptide [1–4]. Extensive research has unveiled not only its anorexigenic effects but also its role as a neuromodulator in various physiological and pathological homeostatic processes [1,5–7]. Current investigations underscore the significant impact of central nesfatin-1 on cardiovascular function [8,9]. Its administration has been linked to hypertension through the activation of sympathetic nerves [10–12]. Our own studies reveal that intracerebroventricular (ICV) application of nesfatin-1 heightens arterial pressure by stimulating peripheral catecholaminergic, vasopressinergic, and renin-angiotensinergic systems [13], as well as the central cholinergic system [14]. The neuropeptide’s presence in cardiovascular-regulating brain region, such as hypothalamus and nucleus tractus solitarius [1–3,15,16], further supports its role in cardiovascular regulation. Despite the absence of a known nesfatin-1 receptor, its cardiovascular effects are modulated by central cholinergic [14], melanocortin [11,17,18], oxytocin [10,18], and corticotrophin-releasing hormone receptors [18].

Similarly, the central lipoxygenase (LOX) and cyclooxygenase (COX) enzymatic paths regulate autonomic modulations such as cardiovascular [19–24], respiratory [23–25], and neuroendocrine systems [19, 26–28]. Products of the central COX pathway act a pivotal role in governing the circulatory system [26, 29–31]. Notably, our prior research indicates that nesfatin-1 enhances the expression of hypothalamic LOX and COX enzymes, leading to increased release of extracellular leukotrienes (LTs) and prostaglandins (PGs) in the hypothalamus [35]. Recent findings further establish that nesfatin-1’s cardiovascular activity is mediated by central COX and LOX enzymes [36]. Despite this, it remains unclear which COX metabolites are responsible for these effects, as suggested by our previous report. To address this gap, our current study aims to explore the impact of central thromboxane (TX) A2, PGE, PGD, and PGF2α—known for their effectiveness in central cardiovascular control—on the MAP and HR impacts induced by ICV administered nesfatin-1.

## 2. Materials and methods

### 2.1. Animals and experimental design

The study was practised at the Experimental Animal Breeding and Research Centre of Bursa Uludağ University, involving adult male Sprague Dawley rats with weights ranging from 255–305 g (n = 70). These rats had ad libitum access to water and pellet food in suitable ambient conditions. Ethical standards were adhered to, with approval obtained from Bursa Uludağ University’s Local Committee of Animal Ethics (2017–13/03).

Each experimental group included 7 rats. Rats in the groups were randomly selected, with each rat participating once in a single experimental group. On each study day, four animals were studied, encompassing both control and experimental groups. The preparation of rats for the experiment commenced at 9 am and concluded at 10 am, with drug administration and cardiovascular recordings initiating at 2 pm.

Surgical procedures were performed under sevoflurane (2%–4%/100% O2) anaesthesia. Initially, catheter (PE50 tubing) containing heparin (100 U/mL) were put into the left arteria femoralis to pursue mean arterial pressure (MAP) and heart rate (HR). Subsequent inserted femoral artery catheter, the guide cannula was placed the right lateral ventricle according to coordinate of 1.0 mm posterior to bregma, 1.5 mm lateral to midline, and 4.2 mm beneath the skull’s surface for ICV drug injection. The guide cannula was affixed to the skull using acrylic cement. The animals were placed on a heated platform during surgery to ensure a consistent body temperature of 37 ^o^C. Postsurgery, rats were housed separately, given 4–5 h for anaesthesia recovery, and displayed no signs of agitation, pain, or discomfort throughout the experiment.

Since we have shown many times in our previous studies that both nesfatin-1 treatment and nesfatin-1 treatment after vehicle pretreatment produce similar cardiovascular effects [13,14,36], the nesfatin-1 produced cardiovascular effects were not demonstrated in present study as a separate set in the current study. Therefore, in the present series of experiments, the central mediation of TXA2, PGF2α, PGE, and PGD in the MAP and HR replies evoked by nesfatin-1 was studied only. For this objective, first the rats were pretreated apart with furegrelate (the TXA2 synthesis inhibitor; 250 μg, ICV), PGF2α-dimethylamine (PGF2α-DMA; the PGF2α receptor antagonist; 10 μg, ICV), AH6809 (the PGE and PGD receptor antagonist; 10 μg, ICV), or vehicle (saline or 5% DMSO; 5 μL, ICV). The rats were treated with saline (5 μL, ICV) or nesfatin-1 (200 pmol, ICV) 5 min after pretreatments. The changes in MAP and HR were enrolled for the subsequent 60 min following nesfatin-1 injection, as the cardiovascular efficacy of nesfatin-1 began to disappear 30 min after ICV injection, according to our previous study data [13,14,36]. Table 1 shows the experimental groups, the n numbers of the groups and the experimental time table in detail. The dose of nesfatin-1 [14, 36], furegrelate [25], PGF2α-DMA, and AH6809 [23] was determined based on our prior studies. The dose of nesfatin-1 selected for the study was determined based on its ability to elicit optimal cardiovascular effects, as observed in comparison to our previous studies [13,14]. The doses of furegrelate [25], PGF2α-DMA, and AH6809 [23] used in the study were chosen because they were the doses that blocked the cardiorespiratory effects of arachidonic acid (AA) in our previous studies.

### 2.2. Measurement of cardiovascular parameters

The enrolment of the MAP and HR parameters was employed MP36 system (BIOPAC Systems Inc., CA, USA). This system was linked to the volumetric pressure transducer (BPT 300, BIOPAC Systems Inc., CA, USA) via the arterial catheter. Monitoring of the rats’ MAP and HR parameters was conducted by analysing the electronic pressure signal within the AcqKnowledge software (BIOPAC Systems Inc., CA, USA). The software autonomously computed HR based on the MAP signal. Blood pressure values were denoted as MAP in mmHg, and HR was stated in beats per minute (bpm).

### 2.3. Drugs and ICV injections

Nesfatin-1 was procured from Sigma-Aldrich Co. (Deisenhofen, Germany), and furegrelate, AH6809, and PGF2α-DMA were obtained from Cayman Chemical Co. (Ann Arbor, MI, USA). Nesfatin-1, furegrelate, and PGF2α dimethylamine were diluted in 0.9% saline, while AH6809 was dissolved in 5% DMSO. The drug solutions were daily prepared as fresh before being implemented to animals. A 10 μL Hamilton syringe attached to PE10 tubing with injection cannula was used to administer the drugs. During the ICV drug injection, the injection cannula was put into the guide cannula, and then the drug as a volume of 5 μL was pushed to lateral ventricle within 60 s. At the end of experiment, the rats were euthanized with an overdose IV injection of pentobarbital sodium following ICV injection of Indian ink and then the brains of all animals used were removed to verify the location of the ICV injection area (data not shown).

### 2.4. Data and statistical analysis

The graphs and table show the data as seven measurements’ means, and standard error of the mean (S.E.M). Data analysis was performed using IBM SPSS Statistics for Windows, version 25.0. The repeated measures analysis of variance (RMANOVA; two-way) was employed, and it was followed by the Bonferroni test as a post-RMANOVA analysis for statistical analysis. The significance level was accepted as p < 0.05.

## 3. Results

**Figure 1:**
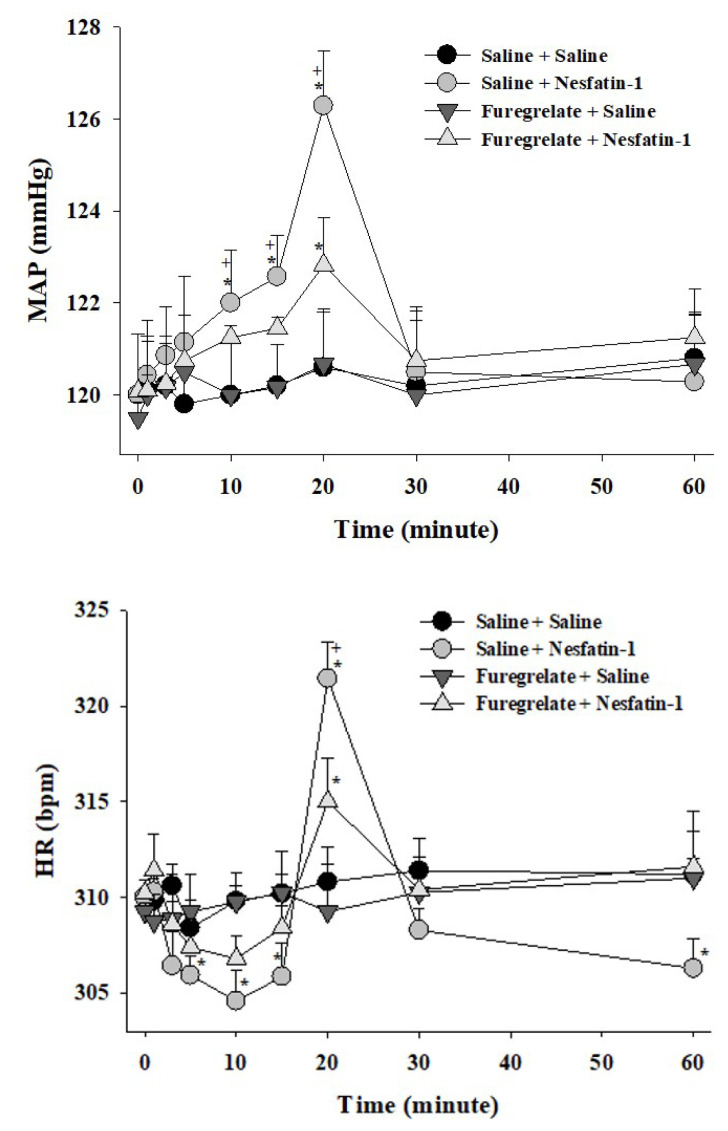
Effect of furegrelate pretreatment on nesfatin-1-evoked cardiovascular responses. Data are given as mean ± S.E.M. (n=7). “0” indicates the time of saline or nesfatin-1 treatment. *p < 0.05 indicates significant difference compared to the “Saline + Saline” or “Furegrelate + Saline” group, ^+^p < 0.05 indicates a significant difference compared to the “Furegrelate + Nesfatin-1” group.

**Figure 2:**
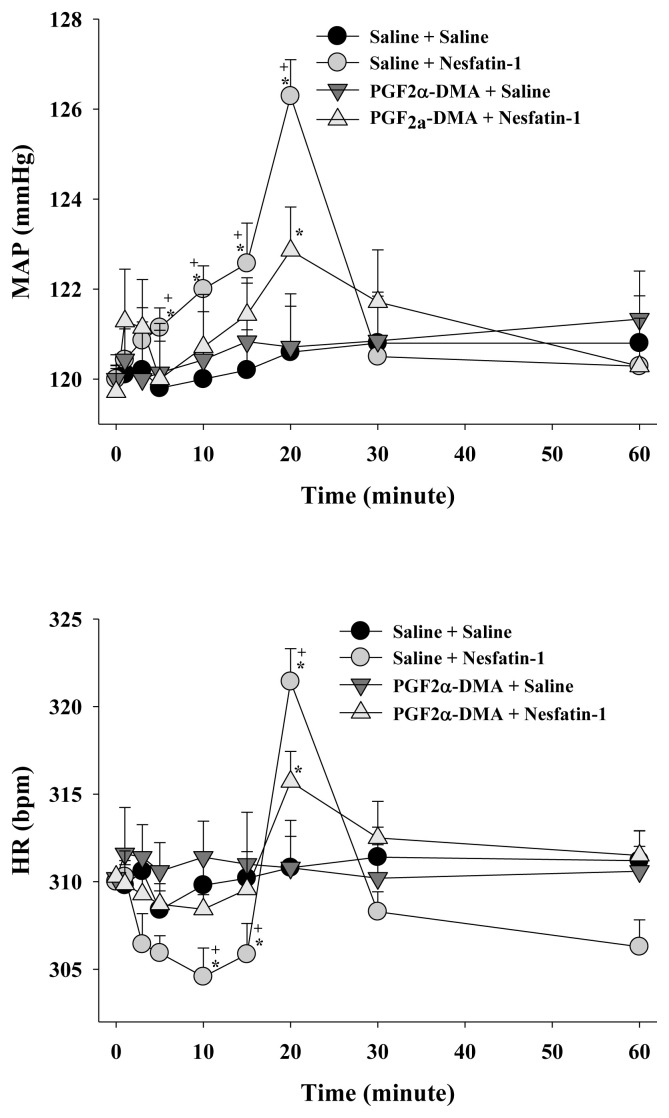
Effect of PGF2α-DMA pretreatment on nesfatin-1-evoked cardiovascular responses. Data are given as mean ± S.E.M. (n=7). “0” indicates the time of saline or nesfatin-1 treatment. *p < 0.05 indicates significant difference compared to the “Saline + Saline” or “PGF2α-DMA + Saline” group, ^+^p < 0.05 indicates a significant difference compared to the “PGF2α-DMA + Nesfatin-1” group.

**Figure 3:**
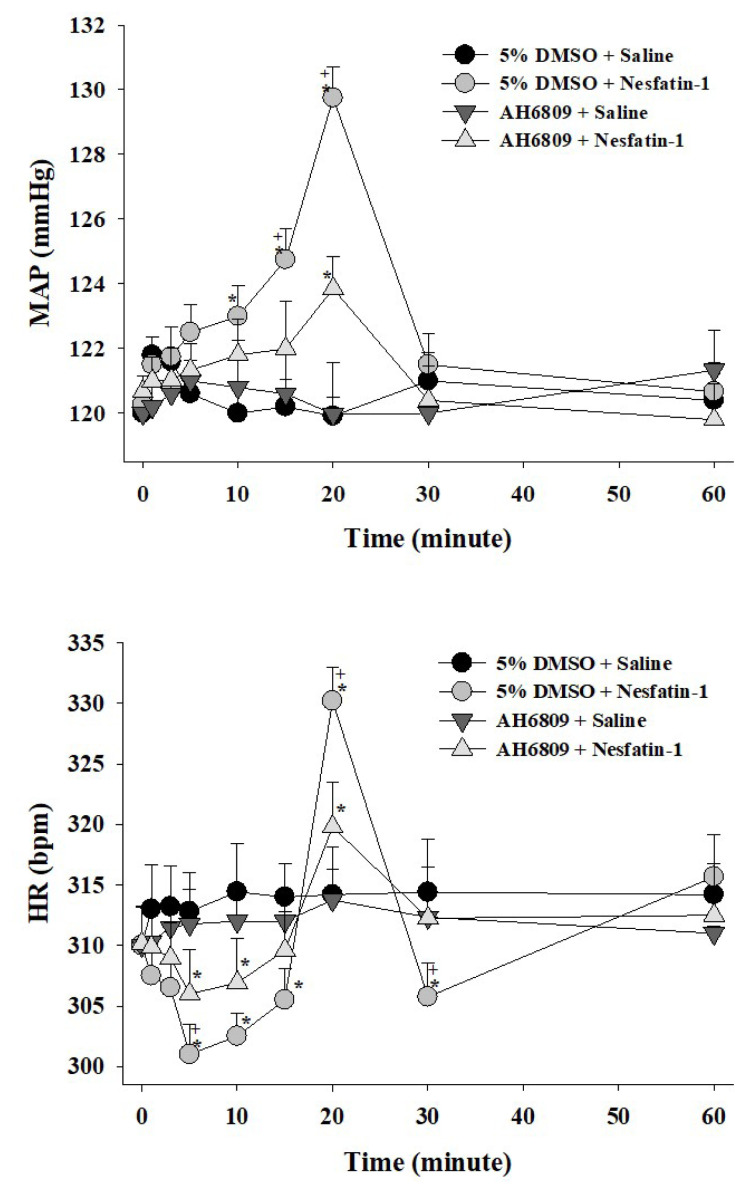
Effect of AH6809 pretreatment on nesfatin-1-evoked cardiovascular responses. Data are given as mean ± S.E.M. (n=7). “0” indicates the time of saline or nesfatin-1 treatment. *p < 0.05 indicates significant difference compared to the “Saline + Saline” or “AH6809 + Saline” group, ^+^p < 0.05 indicates a significant difference compared to the “AH6809 + Nesfatin-1” group.

Centrally administered nesfatin-1 after the vehicle pretreatment (200 pmol, ICV) elicited a notable increase in MAP and induced a heart rate HR response characterized by bradycardic/tachycardic phases (p < 0.05; Figures 1, 2, 3). The elevation in MAP commenced within the initial minutes, reaching a maximum increase of 7.4 ± 1.1 mmHg observed 20 min postimplementation. The elevation in MAP persisted up to 30 min following nesfatin-1 implementation (p < 0.05; Figures 1, 2, 3). Nesfatin-1 also exerted an impact on the heart rate, initiating with bradycardia up to 15 min, followed by tachycardia at 20 min, succeeded by bradycardia again (p < 0.05; Figures 1, 2, 3). The 20^th^ min marked the simultaneous occurrence of the maximum MAP increase and tachycardia (p < 0.05; Figures 1, 2, 3).

The central pretreatment with furegrelate (250 μg), PGF2α-DMA (10 μg), AH6809 (10 μg), or vehicle (5 μL) did not induce alterations in the basal MAP and HR values at the conclusion of the 5-min period, just prior to nesfatin-1 saline (5 μL, ICV) or (200 pmol, ICV) administration (Table 2). Notably, ICV injection of 5% DMSO demonstrated no toxic effects, at least on the cardiovascular system, as evidenced by the absence of a significant difference in MAP and HR responses compared to saline pretreatment (Table 2). Central pretreatment with furegrelate (Figure 1), PGF2α-DMA (Figure 2), or AH6809 (Figure 3) significantly (p < 0.05) abated the MAP and HR responses evoked by nesfatin-1.

## 4. Discussion

The findings underscore that ICV implemented nesfatin-1 exerts a potential influence on cardiovascular parameters, eliciting both pressor and phasic HR responses. Significantly, the data unveil, for the first time, that the furegrelate (the TXA2 synthesis inhibitor), PGF2α-DMA (the PGF2α receptor antagonist), and/or AH6809 (the PGE and PGD receptor antagonist) pretreatments partially inhibit nesfatin-1-evoked cardiovascular responses.

The current dataset illustrates that centrally injected nesfatin-1, following vehicle pretreatment, rapidly induces pressor and biphasic bradycardic/tachycardic heart rate (HR) responses in the cardiovascular system, consistent with our previous findings [13,14,36]. The initial bradycardia observed for the first 15 min, succeeded by tachycardia at 20 min, might be attributed to the rise in MAP following nesfatin-1 injection. This bradycardic response is likely a result of the prompt elevation in MAP activating baroreflex in rats. The subsequent tachycardia at the 20^th^ min of nesfatin-1 application may contribute to the maximal increase in MAP, given the similarity in the time scale of the greatest tachycardic and pressor responses. A noteworthy aspect of the findings is the indication that products of the central COX pathway play at least a partial part in nesfatin-1-eliced cardiovascular effects. This inference arises from the partial blockade of nesfatin-1-evoked pressor and bradycardic/tachycardic cardiovascular responses through central pretreatment with furegrelate, TXA2 synthesis inhibitor; PGF2α-DMA, a nonselective PGF2α receptor antagonist; or AH6809, a nonselective PGE and PGD receptor antagonist. Previously, we reported that nesfatin-1-eliced cardiovascular responses are initially mediated by COX enzyme activation, as evidenced by the complete prevention of these effects in rats pretreated with ibuprofen, a nonselective COX inhibitor [36]. The present study builds on this by suggesting that the subsequent mediators following COX activation in nesfatin-1-induced cardiovascular responses include TXA2, PGF2α, PGD, and PGE, as products of COX. This is supported by the partial blockade of cardiovascular responses with pretreatments involving the furegrelate, the TXA2 synthesis inhibitor, PGF2α-DMA, the PGF2α receptor antagonist, or AH6809, the PGE and PGD receptor antagonist.

AA cascades, characterized by a substantial and impactful turnover rate, serve as neuromodulators or neurotransmitters within the central nervous system [37]. Phospholipase A2 (PLA2) facilitates the release of AA from membrane phospholipids, subsequently giving rise to LTs or PGs via the LOX or COX pathways, respectively [38]. Previous demonstrations from our research indicate that centrally administered PLA2 activator melittin influences the cardiovascular system by activating the central COX- TXA2 signalling pathway, both in normal [27,39,40] and in hypotensive conditions [27]. Moreover, our earlier studies have established that central AA, akin to the PLA2 activator melittin, induces cardiovascular effects by activating central LOX, COX, and/or central COX-TXA2, -PGF2α, -PGD, and/or -PGE signalling paths [19–24]. Central PGE2 [29–31], PGD2 [32,33], PGF2α [30,34], and TXA2 [26] also contribute to the central modulation of the cardiovascular system as subproducts. These reports collectively establish the involvement of the central PLA2-AA-COX or -LOX signalling pathway and their products, PGs and LTs, in cardiovascular regulation. Recent findings from our laboratory demonstrate that central nesfatin-1 treatment activates the central LOX and COX signalling pathways, resulting in elevated release of AA metabolites in the hypothalamus [35]. Additionally, the intercession of central LOX and COX enzymes in nesfatin-1-induced MAP and HR responses has been reported [36]. These earlier reports align with our current findings, suggesting that centrally applied nesfatin-1 increases PG release by activating the central COX pathways. The released cardiovascular active PGs, such as TXA2, PGF2α, PGE, and PGD, may contribute to the observed cardiovascular effects of nesfatin-1. The resemblance between the MAP effect produced by the central PLA2-AA-PGs signalling pathway and the MAP effect following central nesfatin-1 treatment supports the notion that nesfatin-1 may exert cardiovascular effects through increased PGs. Additionally, the partial intercession of nesfatin-1-eliced MAP and HR effects by TXA2, PGF2α, PGE, and PGD, as investigated in the current study, suggests that other COX or LOX metabolites may also play a role in this effect. The bradycardic/tachycardic biphasic heart rate (HR) response observed with central nesfatin-1 administration can be explained by the diverse HR effects of various PGs. Previous reports indicate that centrally applied PGE [29–31], PGD [32,33], and PGF2α [34] induce tachycardic effects, while TXA2 [26] elicits bradycardic responses.

In conclusion, the results affirm that the ICV administration of nesfatin-1 exerts a pressor impact on MAP and induces biphasic HR actions characterized by bradycardia/tachycardia. The key observation in this study is that inhibiting central TXA2 synthesis with furegrelate and blocking central PGF2α and PGD/PGE receptors with PGF2α-DA and AH6809, respectively, partially reverses the MAP and HR responses elicited by nesfatin-1. These findings propose that these COX metabolites may, at least in part, mediate the cardiovascular effects of nesfatin-1. Furthermore, the current data aligns with our last studies on the activation of central LOX and COX by nesfatin-1 [35] and the mediation of central LOX and COX in nesfatin-1-induced cardiovascular responses [36]. This study introduces a new perspective on the central pathways involved in intercession nesfatin-1’s actions on cardiovascular regulation.

## Figures and Tables

**Table 1:** The experimental groups and the experimental timeline.

Experimental groups Pretreatment (dose and route) 5 min Treatment (dose and route)	“n” number
Saline (5 μL; ICV) + Saline (5 μL; ICV)	7
Saline (5 μL; ICV) + Nesfatin-1 (200 pmol; ICV)	7
5% DMSO (5 μL; ICV) + Saline (5 μL; ICV)	7
5% DMSO (5 μL; ICV) + Nesfatin-1 (200 pmol; ICV)	7
Furegrelate (250 μg; ICV) + Saline (5 μL; ICV)	7
Furegrelate (250 μg; ICV) + Nesfatin-1 (200 pmol; ICV)	7
AH6809 (10 μg; ICV) + Saline (5 μL; ICV)	7
AH6809 (10 μg; ICV) + Nesfatin-1 (200 pmol; ICV)	7
PGF_2α_-DMA (10 μg; ICV) + Saline (5 μL; ICV)	7
PGF_2α_-DMA (10 μg; ICV) + Nesfatin-1 (200 pmol; ICV)	7
**Total**	**70**

**Table 2:** The impact of pretreatments on basal MAP and HR parameters of the rats.

Pretreatment	MAP (mmHg)		HR (bpm)	
	Before	After	Before	After
Saline (5 μL)	118 ± 2.21	119 ± 1.93	334 ± 6.72	336 ± 6.14
5% DMSO (5 μL)	120 ± 1.92	121 ± 2.11	328 ± 5.82	330 ± 6.13
Furegrelate (250 μg)	119 ± 2.08	118 ± 2.44	331 ± 6.77	334 ± 7.48
AH6809 (10 μg)	121 ± 1.83	122 ± 1.98	332 ± 7.11	329 ± 6.73
PGF2α-DMA (10 μg)	122 ± 2.24	121 ± 1.72	326 ± 6.38	327 ± 8.12

The measurements obtained just before and five min after the first ICV injections are represented by the values in the table. Data are given as mean ± S.E.M. (n = 7).
